# Isolated Total Rupture of Extraocular Muscles

**DOI:** 10.1097/MD.0000000000001351

**Published:** 2015-10-02

**Authors:** Jingchang Chen, Ying Kang, Daming Deng, Tao Shen, Jianhua Yan

**Affiliations:** From The State Key Laboratory of Ophthalmology, Zhongshan Ophthalmic Center, Sun Yat-sen University, Guangzhou, 510060, People's Republic of China.

## Abstract

Total rupture of extraocular muscles is an infrequent clinical finding. Here we conducted this retrospective study to evaluate their causes of injury, clinical features, imaging, surgical management, and final outcomes in cases of isolated extraocular muscle rupture at a tertiary center in China.

Thirty-six patients were identified (24 men and 12 women). Mean age was 34 years (range 2–60). The right eye was involved in 21 patients and the left 1 in 15. A sharp object or metal hook was the cause of this lesion in 16 patients, sinus surgery in 14 patients, traffic accident in 3 patients, orbital surgery in 2 patients, and conjunctive tumor surgery in 1 patient. The most commonly involved muscles were medial (18 patients) and inferior rectus muscles (13 patients). The function of the ruptured muscles revealed a scale of −3 to −4 defect of ocular motility and the amount of deviation in primary position varied from 10 to 140 PD (prism diopter). Computerized tomography (CT) confirmed the presence of ruptured muscles.

An end-to-end muscle anastomosis was performed and 3 to 5 mm of muscle was resected in 23 patients. When the posterior border of the injured muscle could not be identified (13 patients), a partial tendon transposition was performed, together with recession of the antagonist in most patients, whereas a recession of the antagonist muscle plus a resection of the involved muscle with or without nasal periosteal fixation was performed in the remaining patients. After an average of 16.42 months of follow-up an excellent result was achieved in 23 patients and results of 13 patients were considered as a failure.

In most patients, the posterior border of the ruptured muscle can be identified and an early surgery can be performed to restore function. Alternatively, a partial tendon transposition should be performed. When muscular rupture is suspected, an early orbital CT is required to confirm this possibility, which can then verify the necessity for an early surgical intervention.

## INTRODUCTION

Eye motion limitation and diplopia are commonly observed after a significant injury to the eye and/or orbit. Such abnormalities are usually due to incarceration of extraocular muscles in the orbital fracture or paralysis of oculomotor nerves.^1–3^ Other causes, such as adherent scars, direct injury to extraocular muscles, and orbital hematoma, may also contribute to such strabismus. Ruptures in extraocular muscles can occur after strabismus or after retinal, orbital, or sinus surgeries; however, trauma resulting in a total rupture of extraocular muscles is infrequent in clinical practice.^4–11^ Currently, only single cases or small case series reporting ruptures in extraocular muscles are found in the literature.^12–18^ Such conditions represent a considerable challenge to ophthalmologists with regard to both their timely diagnosis and surgical management.^19–30^ In this report, we present 36 unusual cases of total extraocular muscle rupture as reviewed from the records of a large tertiary ophthalmic center in China over the period of 2003 to 2014. The causes of injury, clinical features, imaging findings, surgical management, and final outcomes are summarized.

## MATERIALS AND METHODS

This retrospective study was performed in compliance with the Declaration of Helsinki, and approved by the ethics committee of the Zhongshan Ophthalmic Center. Data from the medical records of all patients diagnosed with total rupture of extraocular muscles who were referred to the Zhongshan Ophthalmic Center, of Sun Yat-sen University, Guangdong, China, between Jan 1, 2003, and June 30, 2014, were reviewed. All patients were Chinese. The inclusion criteria consisted of (1) no history of previous strabismus or ocular motility disorders and (2) at least 6 months of follow-up examination after strabismus surgery. The exclusion criteria were the patients who showed coexisted eyeball injuries.

A complete ophthalmological examination was performed on each patient and included: visual acuity, intraocular pressure, anterior segment, dilated fundus evaluation, cycloplegic refraction, deviation extent, and motility evaluation. Measurements involving the amount of deviation and evaluation of ocular motility as recorded before and after strabismus surgery were performed by an orthoptist, who was blind as to the surgical treatment of the patient. The prism and alternate cover tests were used to measure the amount of deviation, with the eyes in primary position while fixating with the normal eye at a distance of 6 m. Ocular motility function of each extraocular muscle was clinically evaluated using the standard –4 to +4 grading scale. Forced duction tests were routinely performed. Usually, computerized tomography (CT) or CT in conjunction with magnetic resonance imaging (MRI) of the orbit was used to identify the ruptured muscle and any other structural anomalies of the extraocular muscles before surgical interventions. All surgical complications were recorded and all the surgical procedures were performed by 1 surgeon (JH Yan).

The surgical technique for repair and management of complete muscle loss was based upon the condition of the individual patient. Usually, the trauma was located near the tendon insertion. The 3 most important steps involved with these surgeries involved: (1) identification of the muscle's posterior border, (2) exposure of the muscle fibers, and (3) suture of the posterior border to the anterior border of the muscle. Often 3 to 5 mm of either the posterior or anterior portion of the muscle was resected before the end-to-end muscle anastomosis. When the posterior border could not be identified, in cases where the laceration was distal to the tendon or in the belly of the muscle, a partial tendon transfer from the neighboring rectus muscle to the ruptured muscle area of the globe was performed, together with a recession of the antagonist muscle.

Table 1 presents a summary of the sex, age, involved muscles, surgeries, imaging findings, preoperative and postoperative alignment, and ocular motility, and months of follow-up for each of the patients. Postoperative measurements were performed at the time of the most recent examination. A postoperative horizontal deviation of 0 to 8 PD or a vertical deviation of 0 to 6 PD in the primary position both at distance and near were considered an excellent result. Postoperative results showing a horizontal deviation of >8 PD or a vertical deviation of >6 PD were considered as failed outcomes.

**TABLE 1:**
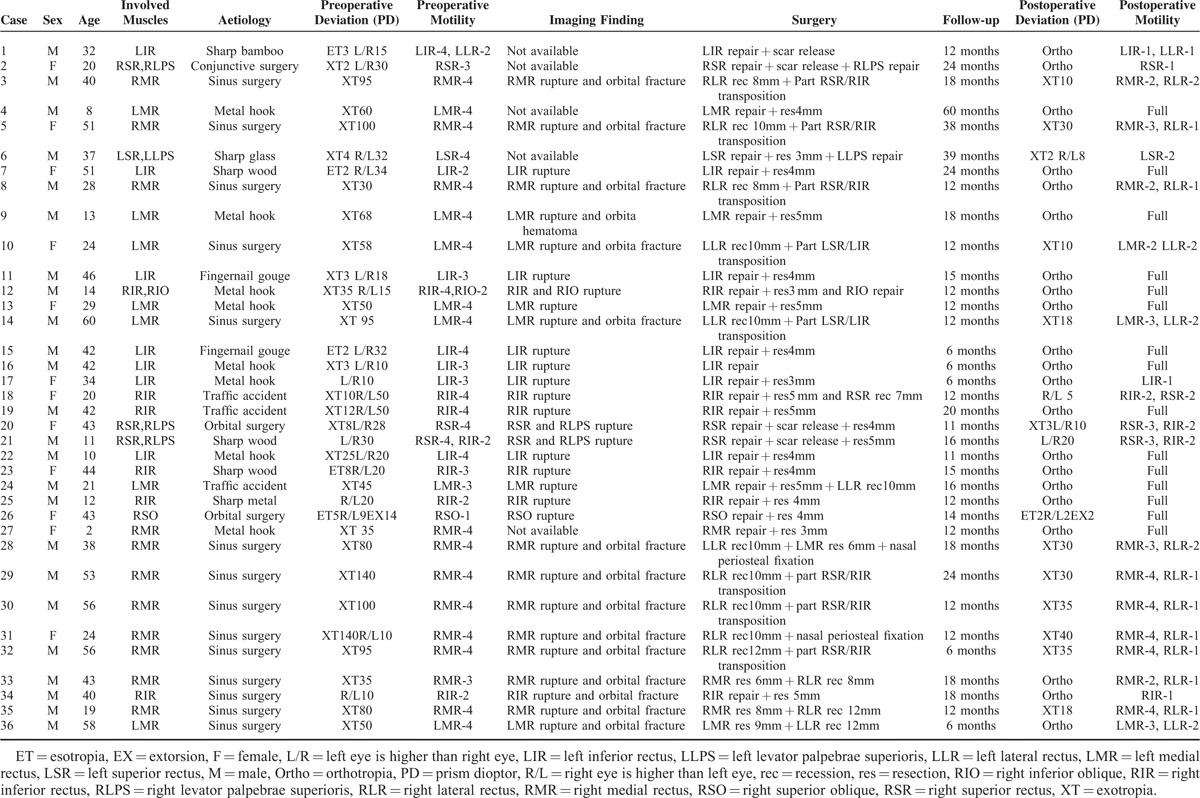
The Surgical Results in 36 Patients With Extraocular Muscle Rupture

## RESULTS

Thirty-six patients with total rupture of the extraocular muscle, who met inclusion criteria, were identified (24 men and 12 women). Mean age was 34 years (range 2–60). The right eye was involved in 21 patients and the left in 15 patients. The causes and other clinical findings of the muscle rupture are shown in Table 1. A sharp object or metal hook was the cause in 16 patients, sinus surgery in 14 patients, traffic accident in 3 patients, orbital surgery in 2 patients, and conjunctive tumor surgery in 1 patient. The most commonly involved muscles were medial (18 patients) and inferior rectus muscles (13 patients). Both the inferior rectus and inferior oblique muscles were affected in 1 patient. The superior rectus muscle was involved in 4 patients, together with involvement of the levator palpebrae superioris. The superior oblique was involved in only 1 patient. All together, 41 muscles (including 4 levator palpebrae superioris muscles) were involved in this study.

All patients had disturbance of ocular motility and diplopia at presentation and none showed spontaneous recovery of motility defects during the period between muscle injury and their initial presentation. However, the amount of deviation in primary position changed in 12 patients, among whom 5 patients increased >10 PD and 7 patients decreased >10 PD. The time interval between muscle injury and surgical intervention ranged from 4 h to 10 months with an average of 96 days. The function of all the ruptured rectus muscles revealed a scale of −3 to −4 defect, but in cases 7, 25, and 34, a scale of −2 defect of the inferior rectus muscles was present, whereas functioning of the ruptured oblique muscles showed a scale of −1 to −2 defect (cases 12 and 26). As a result of a scar formation around the involved rectus muscle, cases 1 and 21 also had a scale of −2 motility defect of the lateral rectus and inferior rectus muscles. The amount of preoperative deviation in primary position varied widely, from a deviation of 10 to 140 PD, with an average of 50 PD. CT or MRI was available in 31 patients. All 31 patients demonstrated a ruptured muscle; 14 patients subjected to sinus surgery showed medial wall fractures (13 cases), whereas a medial-inferior wall fracture was present in the remaining case of the involved orbit. One patient (case 9) had remarkable orbital hematoma at the site of the injured medial rectus muscles.

After diagnosis of traumatic rupture of the extraocular muscles, repairs of the muscles were performed in 23 patients. The muscles were retrieved by means of a routine conjunctival approach in 19 patients and by a transcutaneous superior orbitotomy approach in 4 patients. Usually, the trauma was located within 3 to 8 mm to the tendon insertion and the posterior border was surrounded by Tenon's capsule tissue. Depending on the degree of muscle injury, an end-to-end muscle anastomosis and resection at 3 to 5 mm of either the posterior or anterior portion of the muscle was performed. In 13 patients with sinus surgery it was not possible to identify the posterior border of the injured muscle as the muscle rupture was either too distant from the tendon or located in the belly of the muscle. In these patients, a partial tendon transposition from the neighboring rectus muscle to the ruptured muscle together with a recession of the antagonist was performed in 8 cases; a recession of the antagonist plus a resection of the involved muscle was performed in 4 cases; or a recession of the antagonist plus nasal periosteal fixation was performed in 1 case.

**FIGURE 1:**
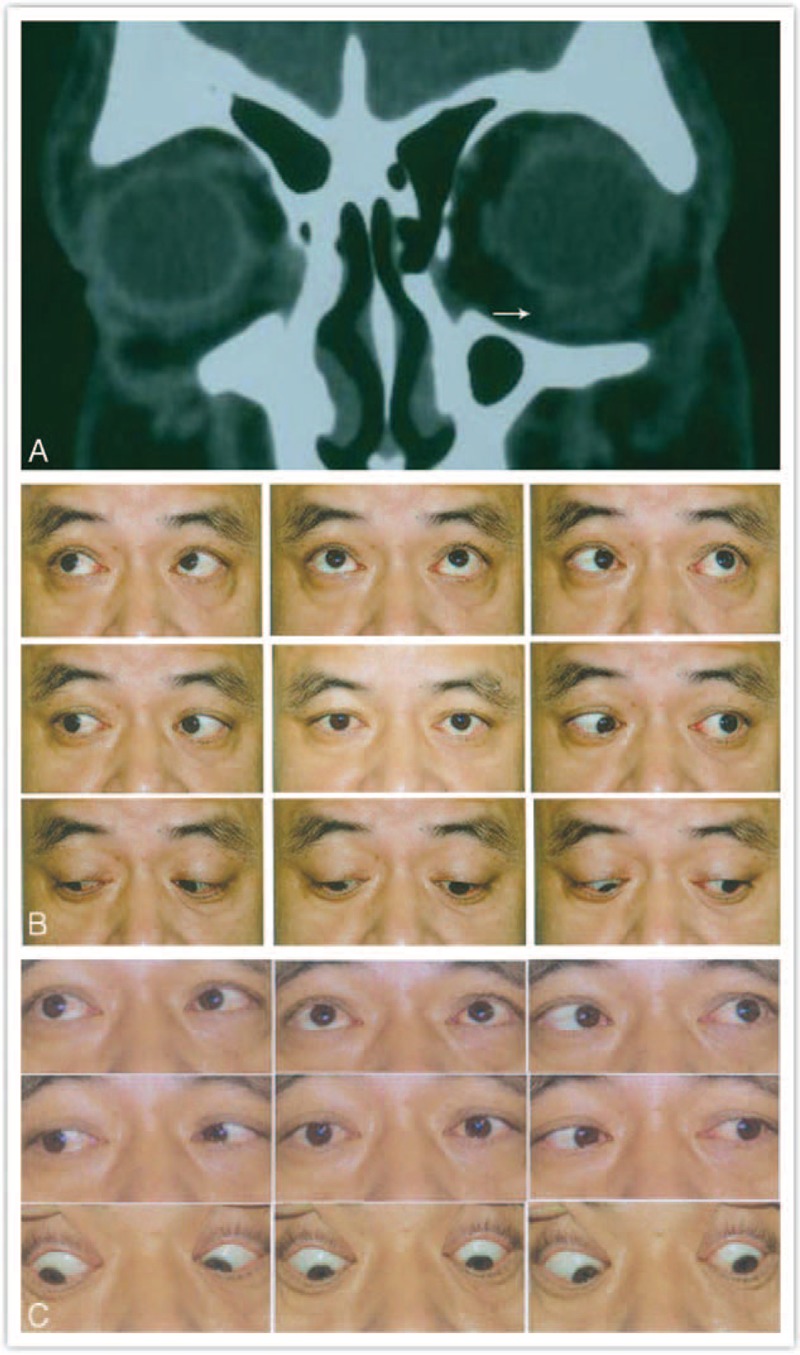
A–C: Preoperative and postoperative alignment and CT of case 11 with a total rupture of the left inferior rectus muscle. A: Orbital CT showing an impinged left inferior rectus muscle. B: Before surgery, 18 PD left hypertropia and a scale of −3 motility defect of left inferior rectus muscle, together with left lower eyelid retraction. C: 15 months after surgical repair (end-to-end muscle anastomosis and a 4 mm resection of the ruptured inferior rectus muscle), orthotropia, and normal ocular motility. CT = computerized tomography, PD = prism diopter.

**FIGURE 2:**
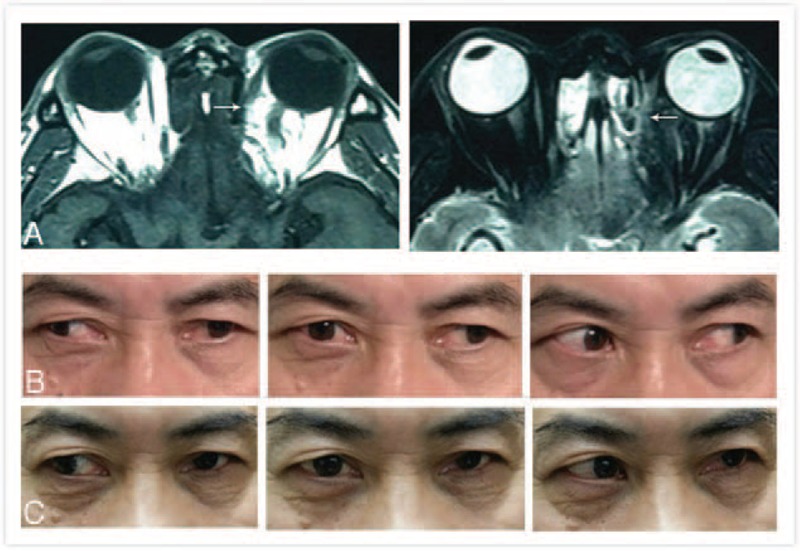
A–C: Preoperative and postoperative alignment and magnetic resonance imaging (MRI) of case 36 with a total rupture of the left medial rectus muscle after sinus surgery. A: MRI in both T1WI and T2WI demonstrates the total rupture present in the left medial rectus muscle. B: Before surgery, the patient had a 50 PD left exotropia and a scale of −4 motility defect of left medial rectus muscle. C: 6 months after strabismus surgery (9 mm resection of the ruptured medial rectus muscle and 12 mm recession of the antagonist lateral rectus muscle), orthotropia in primary position and a scale of −3 and −2 motility defect of both the left medial rectus and lateral rectus muscles, respectively. MRI = magnetic resonance imaging, PD = prism diopter. .

After an average of 16.42 months of follow-up, an excellent result was achieved in 23 patients, whereas results from 13 patients were considered a failure. Of the 23 successes, 15 had normal ocular motility, 5 showed mild motility problems, and 3 had moderate/severe motility defects after successful end-to-end muscle anastomosis (Figure 1). However, all 13 patients with nose surgery had persistent motility defects ranging from moderate to severe after strabismus surgery, during which the posterior border of the muscle could not be identified (Figure 2).

## DISCUSSION

Direct extraocular muscle contusion, nerve damage, and orbital fracture are usually the major causes of posttraumatic strabismus and diplopia. In order to avoid a potential functional sequellae, it is important to promptly determine whether an orbital fracture is present.^1,3^ In the absence of a fracture, strabismus surgery is usually delayed for >6 months, even when the extraocular muscle or nerve damage is relatively severe as spontaneous improvement of the strabismus and diplopia may result. However, extraocular muscle rupture can be present without orbital fractures. For example, a history of nasal surgery or the presence of a sharp object impacting on the eye can result in extraocular muscle rupture and therefore should alert clinicians to the potential of this diagnosis. When the presence of an extraocular muscle rupture is suspected, surgical exploration should be immediately considered in order to avoid persistent strabismus and diplopia.^1–5^ A CT should be mandatory in such cases for the identification of both an orbital wall fracture and laceration of extraocular muscles. When the CT is normal in cases with persisting diplopia, MRI can be very useful for revealing the presence of severed muscles.^2,17,18^ Although the injury history, performance of eye/nose surgery, clinical features, and orbital imaging are all useful in identifying a ruptured muscle, no single clinical finding provides an absolutely reliable means for determining a muscle rupture.^5^ The oculocardiac reflex represents another diagnostic test that can be applied for identifying lost muscle during surgery. All patients in this study had disturbance of ocular motility and diplopia at presentation and functioning of the ruptured muscles in most (31/36–86%) revealed a scale of −3 to −4 defect. As a result of scar formation around the involved rectus muscle, 2 patients (cases 1 and 21) also had a scale of −2 motility defect of the adjacent extraocular muscle (lateral rectus muscle) and the antagonist (inferior rectus muscle). The amount of deviation in primary position varied from 10 to 140 PD, with an average of 50 PD.

When extraocular muscle injury results from trauma, it usually is associated with extensive orbital and ocular damage.^23^ Extraocular muscle rupture without eye damage is infrequently encountered. Diplopia, strabismus, and compensatory head posturing are distressing for patients from both cosmetic and functional considerations. All 6 extraocular muscles might be involved in these conditions.^5,23–27^ However, the most commonly involved muscles are the medial and inferior rectus muscles, as these muscles are nearest to the corneoscleral limbus. In addition, Bell's phenomenon, a protective upward and outward movement of the eye, increases the exposure of these 2 muscles.^2–8^ Of the 36 cases (41 muscles) reviewed in this study, the medial rectus was involved in 18 patients and inferior rectus in 13 patients, findings which were similar to that of previous reports.^12–20^ A sharp object or metal hook seems to have been the cause of this lesion in these patients. In this study, a sharp object or metal hook were the cause of the lesion in 16 patients, nasal surgery in 14 patients, and orbital/conjunctive tumor surgery in 3 patients. For only 3 patients was the cause identified as a traffic accident; however, invasion of a sharp object due to the accident might have been involved in these cases. Men (2:1) and the right eye (7:5) were more frequently involved, suggesting that the increased amount of activity of men and a dominance of right-handedness may be related to these findings.

The repair and surgical management of traumatic or surgical muscle loss is very difficult.^19–22^ Two major goals to be accomplished by such surgery include an assurance for fusion in the primary position and a favorable cosmetic outcome. Selection of a specific surgical approach for repairing a lost extraocular muscle should be made on an individual patient basis. In blunt or sharp trauma, the muscle may be completely avulsed or be cut from its insertion, without losing its attachments to the intermuscular septum. Such an approach allows the surgeon to relocate the muscles with relative ease. Usually, the trauma is located at or near the tendon insertion and the damaged muscle is almost always located at or near its penetration site.^2^ In such cases, it is necessary to first identify the posterior border, which was usually surrounded by Tenon's capsule, then expose the muscle fibers, and finally suture the posterior border of the muscle to its anterior border with a nonabsorbable synthetic suture. We have found these to be the most important 3 steps involved with this difficult procedure. Slipped muscles can be retrieved by following the thin avascular muscle capsule posteriorly until the muscle is identified. A lost muscle can be found using a traditional conjunctival approach, using either an external orbitotomy or an endoscopic transnasal method.^5,19^ When the anterior border fails to include a viable portion of muscle, the posterior border should be sutured to the sclera where its original anatomical position was located. We often resected 3–5 mm of either the posterior or anterior portion of the muscle before the end-to-end muscle anastomosis. In cases where it is difficult to perform muscle anastomosis due to the presence of large gaps between the posterior and the anterior borders, a “hang-back” suture technique, combined with recession of the ipsilateral antagonist, is advisable.^11,23^ However, when the laceration occurs distal to the tendon or in the belly of the muscles, surgical repair becomes more difficult.

When the posterior border cannot be identified, the antagonist muscle is weakened and a transposition of adjacent muscles is performed.^5,6,10^ Muscle loss following sinus surgery usually involves a significant amount of tissue loss and a muscle length defect, by eroding a portion of the muscle. In such cases direct repair is not possible. Nonabsorbable sutures may be used to bridge this gap. If the sutured muscle does not recover its function or diplopia and strabismus persist, the possibility of nerve damage requires consideration. Injections of botulinum toxin into the ipsilateral antagonist provides a useful means of assessing this potential.^2,20^ Subsequently, a muscle transposition should also be considered within the first 4 to 6 months after the first surgery. Jensen's transposition procedure serves as an effective approach to correct the deviation in the primary position, under conditions where the patient has sufficient scar formation around the ruptured muscle to tolerate Jensen's operation. Otherwise, a modified Jensen transposition procedure, or a muscle transposition without disinsertion can be used.^21^ Finally, prisms are useful to treat small deviations of concomitant tropias. In this study, an excellent result was achieved in 23 patients, whereas 13 cases were considered failures following strabismus surgery, indicating an intact oculomotor nerve and good fusion. Patients usually regain fusion after muscle repair. In all 13 patients with nasal surgery, persistent moderate or severe motility defects were present, when the posterior border of the muscle could not be identified.

Our study differs from that of previous reports in several respects: (1) a sharp object/metal hook and sinus surgery were the main cause of the lesions reported here, (2) we often resected 3 to 5 mm of either the posterior or the anterior portion of the ruptured muscle before the end-to-end muscle anastomosis, and (3) in addition to the use of surgical techniques for muscle transposition, a recession of the antagonist plus a resection of the involved muscle were also used to correct the deviation when it was impossible to repair the ruptured muscle, under conditions where sufficient scar formation was found around the ruptured muscle.

The limitation of our study is that it was a retrospective study, which was subject to measurement and interpretation errors. Also it was difficult to accurately compare the effect of different surgical methods when dealing with patients who showed different history of trauma and various clinical features. The follow-up time was relatively short and quite variable among individual cases. Finally, our hospital mainly enrolled patients from south China, which could introduce some geographical bias in our series.

In conclusion, we report a relatively large case series of extraocular muscle ruptures in Chinese patients resulting from both trauma and surgery. Due to the remarkable strabismus, diplopia, defects of ocular motility in the direction of the suspected rectus rupture, and observations from CT or MRI imaging, an immediate surgical intervention was performed in these patients. The posterior border of the ruptured muscle was identified and the posterior and anterior borders could be sutured in most patients. A 3 to 5 mm resection of the ruptured muscle was usually performed in these damaged muscles. After surgery, ocular motility was fully or partially restored and diplopia absent or remarkably reduced. When it is not possible to identify the posterior border, a partial tendon transposition surgery and recession of the antagonist should be performed. When muscular rupture is suspected, an early orbital CT or MRI is required to confirm this possibility, which can then verify the necessity for an early surgical intervention.
